# Supplementary material for: The DmeRF System Is Involved in Maintaining Cobalt Homeostasis in Vibrio parahaemolyticus
Source: Int J Mol Sci. 2022 Dec 27;24(1):414. doi: 10.3390/ijms24010414 (PMC9820535; doi:10.3390/ijms24010414)
Supplement: Supplementary file 1 [file ijms-24-00414-s001.zip › ijms-2017428-supplementary.pdf]

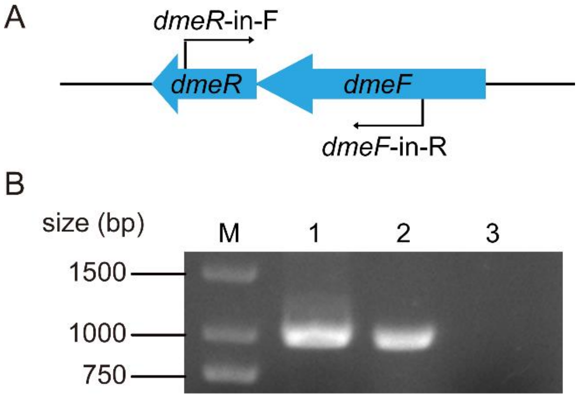

Figure S1. Co-transcription analysis of the *dmeR* and *dmeF* genes. The locations of the primers used for co-transcription analysis (i.e. *demR*-in-F and *dmeF*-in-R) are indicated in (**A**). (**B**) PCR was carried out using using genomic DNA (lane 1), cDNA generated from total RNA of RIMD 2210633 (lane 2), and cDNA– (cDNA reaction without reverse transcriptase). Lane M indicates the DL 2000 DNA Marker.

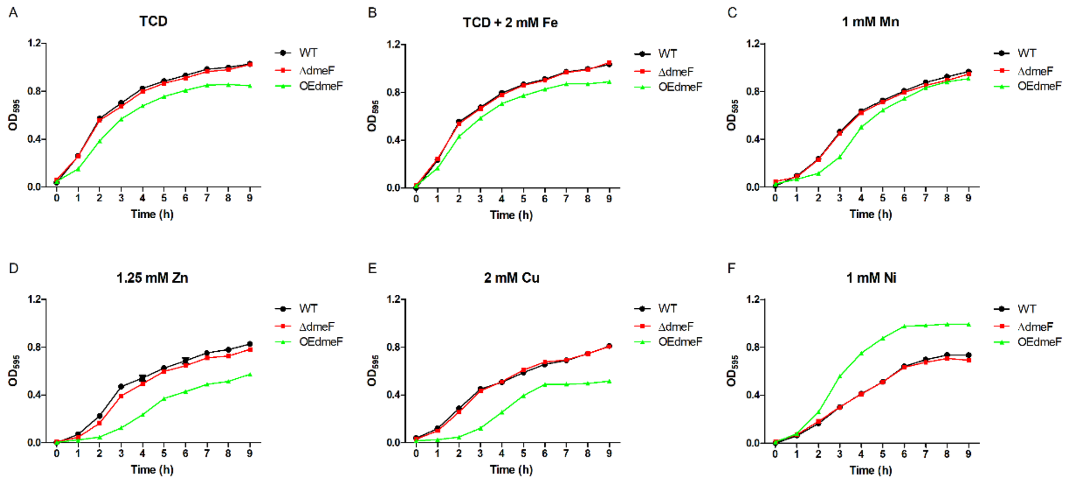

Figure S2. Growth curves analysis of the *V. parahaemolyticus* strains in the presence of various metals. The strains were grown in trisodium citrate dihydrate (TCD, as a control for the Fe[II] treatment) (**A**), 2 mM Fe(II) (**B**), 1 mM Mn (**C**), 1.25 mM Zn (**D**), 2 mM Cu (**E**), or 1 mM Ni (**F**). The experiments were performed at least three times; the results represent the means and SD from three wells in a representative experiment.

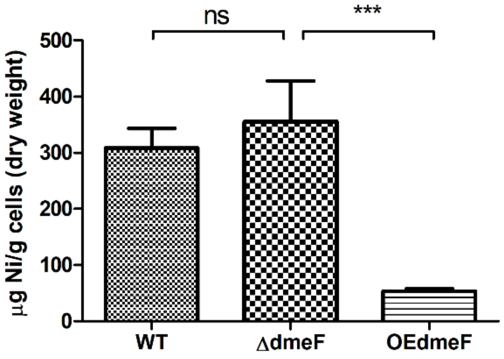

Figure S3. Cellular Ni content in the WT, Δ*dmeF*, and OE*dmeF* strains. These strains were grown in the presence of 1 mM NiSO4 for 6 h. Cellular Ni content was analysed by inductively coupled plasma-mass spectrometry. Results represent the means and SD from five biological replicates. The data were analysed using one-way analysis of variance along with Bonferroni's post-test. ns, not significant; ***, $p < 0.001$.

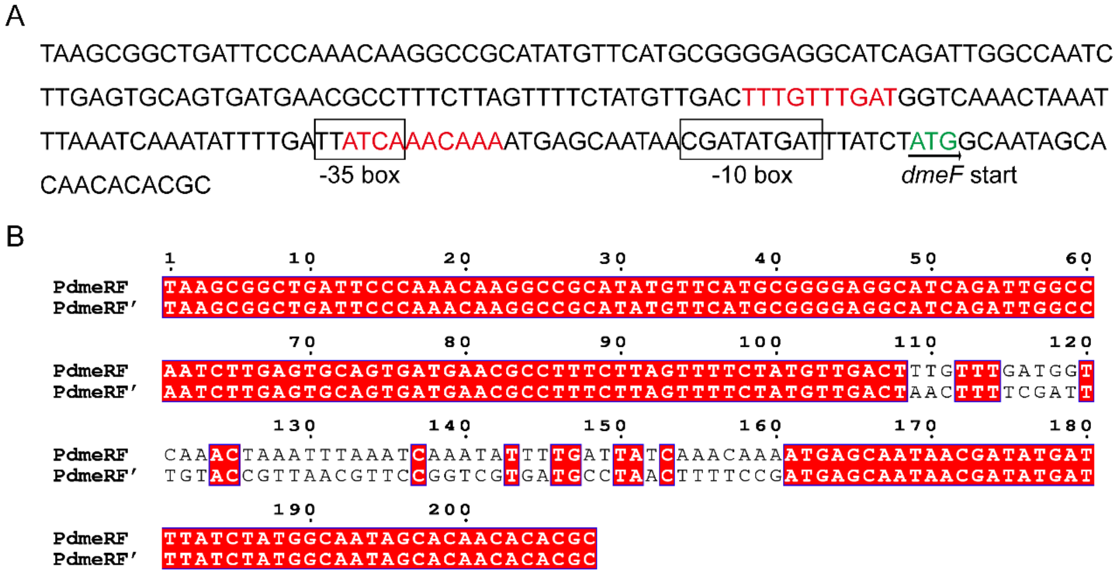

Figure S4. Mutation of the promoter of the *dmeRF* operon. (**A**) The promoter of the *dmeRF* operon. The ATG start codon for DmeF is shown in green letters. The putative −35 and −10 regions are boxed. The sequence flanked by the two inverted repeats (shown in red letters) is the predicted binding site for DmeR. (**B**) Sequence alignment of the WT and mutant promoters. The promoter of *dmeRF* was mutated by replacing the putative binding site for DmeR with an unrelated DNA sequence, to generate P_{dmeRF}'.
